# Can artificial intelligence create human touch in medical writing? A pilot study

**DOI:** 10.3325/cmj.2026.67.66

**Published:** 2026-04

**Authors:** Shigeki Matsubara

**Affiliations:** 1 Department of Obstetrics and Gynecology, Jichi Medical University, Tochigi, Japan; 2 Department of Obstetrics and Gynecology, Koga Red Cross Hospital, Koga, Japan; 3 Medical Examination Center, Ibaraki Western Medical Center, Chikusei, Japan

## Abstract

**Aim:**

To assess whether ChatGPT can autonomously generate and select “human touch” elements (anecdotes, beliefs, and old sayings) and produce writing comparable to human-authored manuscripts.

**Methods:**

I composed a disagreement letter and then tasked ChatGPT-5 with writing a new disagreement letter. The model was instructed to select suitable anecdotes from a candidate list and generate new ones. Both letters were compared. Eight experienced researchers independently assessed whether the letters were appealing.

**Results:**

ChatGPT was able to select appropriate elements from the candidate list and, importantly, generate new ones. The human-generated letter was found to be more appealing by five of eight reviewers, and the ChatGPT-generated letter by three reviewers. None of the researchers reported that they found the use of human touch inappropriate or disruptive.

**Conclusion:**

Although a single case was studied, these findings may help inform reflection on the use of LLMs in medical writing.

Large language models (LLMs) in medical writing have become a high-interest topic (1,2). They offer a valuable opportunity to reconsider the nature of human writing. The issue of “human touch” in ChatGPT-generated medical writing has been discussed previously (3). It was suggested that human touch, including personal anecdotes, beliefs, and old sayings, enriches medical writing (4-6). It was also suggested that, as LLMs rarely access such anecdotes, this element may characterize human writing (3,4). The importance of human judgment in determining whether to incorporate such human touch has also been discussed from a conceptual perspective (7). Yet, with careful input of human touch elements, ChatGPT-generated manuscripts can appear equally appealing (3,6), blurring the distinction.

This pilot study aimed to answer whether ChatGPT can “generate” appropriate human touch. As a prerequisite to addressing this question, I also tested whether ChatGPT can “select” the most suitable human touch elements from multiple candidates and whether such a ChatGPT-generated manuscript appealed to readers.

## Methods

The primary aim of this study was to determine whether ChatGPT was able to “generate” some elements of human touch, and the assessment of ChatGPT’s ability to “select” the elements of human touch served as preparatory work. However, generally speaking, “selection” may be an easier task for both ChatGPT and human authors, and “generation” may come after appropriate selection behavior.

**Figure 1:**
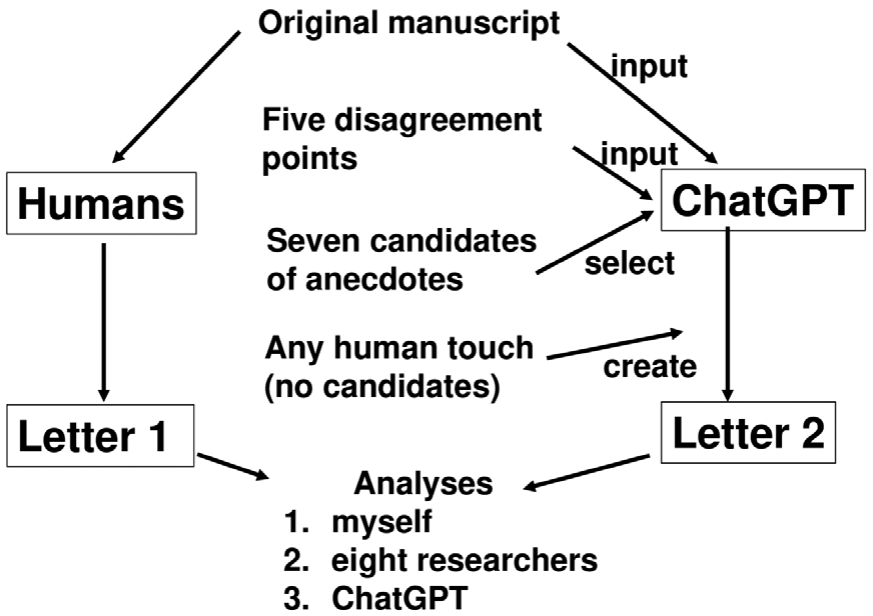
The study design.

Eight experienced researchers evaluated whether either letter appealed more to them. I also asked ChatGPT to assess the likelihood of journal acceptance for both letters. I reset ChatGPT before each step to extinguish ChatGPT’s memory. Fundamental study design is shown in Figure 1.

First, I wrote a disagreement letter addressing an article (8) claiming that the clinic doctor who transferred the patient could have been a coauthor of the case report. Five “disagreement points” (Box 1) formed the basis of Letter 1 (Box 2). This article had also been used in my previous experiment with a similar design (5). The “double use” was intentional: in the earlier study, I examined how ChatGPT-generated manuscripts appealed to readers, without focusing on “human touch” (5). Using the same article and design allowed me to focus more directly on this aspect.

Box 1 Instructions given to ChatGPT for writing a disagreement letter*

**Task:** Draft a Disagreement Letter addressing the original article provided.

i) Include all five “Disagreement Points” indicated in (A).

ii) Incorporate at least one or more “Anecdotes” from (B). Select those that are relevant to the context of the Disagreement Letter.

iii) You may craft and include additional elements of human touch (eg, anecdotes, proverbs, or sayings) only if they enhance the context of the Letter. There is no need to forcibly create new “human touch” elements.

A) Disagreement points:

1. Determining authorship is very difficult even within a university department that cares for transferred patients, let alone for clinic doctors who transfer patients.

2. If the notion that “a clinic doctor transferring a patient should be an author” becomes a norm, it could lead to gift authorship.

3. In the university-clinic relationship, there may be “within-area power balances,” where one clinic doctor is given more weight than another. This could influence decisions about clinic doctors’ co-authorship.

4. How much clinic doctors value co-authorship is doubtful; they may not value it at all.

5. Therefore, I believe that the current practice, where clinic doctors usually do not become coauthors, works well and is justified.

B) Candidate anecdotes

1) When I see my name listed under “Reviewers this year” in a journal, I don’t feel happy; mere name recognition is not important to me.

2) An old friend, a clinic doctor, said, “Now I am free from the ‘publish or perish’ atmosphere, which gives me peace of mind.” A clinic doctor may not gain much merit from being a coauthor and might not even welcome it.

3) A friend in the film industry told me, “Seeing my name at the end credits doesn’t make me happy.

4) A former editor-in-chief once remarked, “Too many authors in a case report generally feels awkward.”

5) An old professor observed, “The more authors there are, the lighter each coauthor’s contribution seems.”

6) The first Nobel Prize^†^ in Physiology or Medicine in 1901 was awarded to Von Behring, with Shibasaburo Kitasato excluded. Some argue this was partly because Von Behring’s 1890 paper was single-authored without including Kitasato. While co-authorship can occasionally be significant, such cases are very rare.

7) Rosalind Franklin was not listed as a coauthor in the 1953 *Nature* paper on DNA structure, but her contribution was crucial, leading to the Nobel Prize for Watson and Crick: coauthorship is not always important.

*ChatGPT-generated letter (Letter 2) incorporated anecdotes 2), 4), and 5) while excluding 1) and 3). For 6) and 7), the reference to the “Nobel Prize” ^†^ was included without detailing Kitasato or Franklin.

Box 2 Human-written and ChatGPT-generated letters*

**Letter 1 (human-written letter)**

Clinic doctor authorship in case reports: The current practice works

The author demonstrated that there are very few case reports where a clinic doctor transferring the patient becomes a coauthor of a case report. He claimed that a clinic doctor, if contributing, should be listed as a coauthor: this will strengthen university-clinic “cooperation.” I respectfully disagree with the author’s claim.

First, contribution to the paper is difficult to evaluate. During my tenure as a university director, I often encountered questions of “who contributed” in case reports: every department member “cared and considered” and thus contributed. There may be 100 clinic doctors behind 100 case reports. “Contribution as coauthor” is difficult to determine.

Second, as in any society, hidden power dynamics among clinic doctors can complicate authorship decisions. For example, one clinic doctor might be a respected former professor, while another is a newcomer. If such dynamics influence coauthorship, it risks academic inequity. Additionally, if clinic doctors being coauthors becomes a “norm,” gift authorship could become an issue. This risks creating “mal-relationships” rather than fostering “good cooperation.”

Third, some clinic doctors may prioritize clinical practice over academic publication, finding satisfaction in their direct patient care rather than in coauthorship.

Some argue that Shibasaburo Kitasato, not being listed as a coauthor in Behring’s 1890 paper, lost the chance to receive the first Nobel Prize in Medicine in 1901. While I understand the importance of authorship, Matsubara’s context may apply in such critical academic situations. If coauthorship determination can be made fair and mutually beneficial, I would welcome Matsubara’s proposal. However, these conditions are not yet a reality. Thus, I consider that the current practice, where clinic doctors usually do not become coauthors, works well and is justifiable. The author’s proposal, while well-intentioned, risks creating more problems in real-world practice.

**Letter 2: ChatGPT-generated letter**

Coauthorship in academic publishing: Quality over quantity

I respectfully disagree with the author’s suggestion that clinic doctors transferring patients should be considered as coauthors. Determining authorship is already challenging within university departments handling transferred patients, and this complexity increases when involving clinic doctors.

Institutional hierarchies and power imbalances within regions could unduly influence decisions on coauthorship, potentially favoring some clinic doctors over others. If inequity arises among clinic doctors, it might hinder university-clinic cooperation, or even clinic-clinic cooperation, in the corresponding area. Furthermore, the risk of promoting gift authorship—a practice detrimental to the integrity of academic publishing—cannot be ignored.

Coauthorship is undoubtedly meaningful in some contexts; an extreme example may be seen in the Nobel Prize selection process. However, I question how much clinic doctors value coauthorship in practice. A clinic doctor once remarked, “Now I am free from the 'publish or perish' atmosphere, which gives me peace of mind.” This sentiment may reflect a broader reality: although evidence is lacking, many clinic doctors might not view coauthorship as a professional necessity or even as a source of satisfaction.

Moreover, the inclusion of numerous coauthors can dilute the perception of individual contributions. I, as a former editor-in-chief of a medical journal, once observed, “Too many authors in a case report generally feels awkward.” Coauthorship should reflect substantial contributions rather than serve as a symbolic gesture to enhance the appearance of collaboration.

As far as coauthorship is concerned, it’s not about ‘the more, the livelier the atmosphere’; substance matters more than numbers. Adding coauthors for variety alone risks undermining the essence of genuine contribution, much like how even a mountain crowded with trees gains nothing from lifeless trunks^†^—an overstatement, of course,^§^ expressed in a manner akin to a Japanese proverb.

*Both letters contain 284 words, which is a coincidence. ChatGPT created proverb (human touch)^†^ without being given any candidates, and, furthermore, it softened the expression.^§^

Second, I tasked ChatGPT-5 with generating a disagreement letter (Letter 2, Box 2) using three inputs: i) the paper (8), ii) the five disagreement points, and iii) seven candidate anecdotes (Box 1). ChatGPT was instructed to select suitable human touch elements (excluding unsuitable ones) and, importantly, to freely add new ones, being allowed to “generate new anecdotes.”

Third, two letters were compared in three different ways:

1) I compared the two letters, focusing on their human touch.

2) Eight experienced Japanese physician-researchers, each with 21-38 years (median: 27) of clinical practice and 31-336 (median: 81) PubMed-indexed papers, evaluated which letter was more “appealing.” They were not informed that the study concerned ChatGPT or “human touch.” They were blinded to the origin of the two letters. The two letters were presented in the same format (Box 2). To minimize the “order effect” (ie, the first-read letter may be perceived more favorably or unfavorably), four reviewers received Letter 1 followed by Letter 2, whereas the remaining four received Letter 2 followed by Letter 1. Reviewers were not asked to evaluate “human touch” elements, as doing so might have alerted them that the study concerned AI-generated manuscripts. Such awareness could have shifted their attention toward identifying which letter was AI-generated, thereby potentially biasing their evaluation. This study did not aim to determine the overall superiority of the two letters (human- vs AI-generated). Rather, the evaluation was intended as a minimal prerequisite – to ensure that the inclusion of “human touch” did not diminish the overall appeal of the letter. Accordingly, reviewers were simply asked which letter was more “appealing,” and this deliberately broad question was chosen to avoid introducing bias. This approach, however, allows for the possibility that factors other than “human touch” may have influenced reviewers’ impressions.

3) I asked ChatGPT-5 to assess which letter was more likely to be accepted by a general medicine journal. This estimation of “acceptance likelihood” functioned as an informal indirect measure of overall appeal.

## Results

Both letters covered all five “disagreement points.” The human-generated letter, written in simpler English, was somewhat informal, whereas the ChatGPT-generated letter was more formal; the differences were minimal. Both included “human touch” elements, with their degree and placement judged appropriate. Notably, ChatGPT “selected” certain anecdotes, using some and excluding others from the seven candidates. Importantly, it generated and added an “old saying.” ChatGPT generated this element without being provided with any specific candidates.

Five out of eight reviewers found the human-generated letter more appealing. Four reviewers noted that the anecdotes enhanced readability and persuasiveness – one favoring Letter 1 and three favoring Letter 2. Importantly, none considered the human touch distracting. Letter order (ie, which letter was read first) did not affect the results.

ChatGPT judged both letters were equally likely to be accepted by a journal. Box 3 presents ChatGPT’s evaluation.

Box 3 **ChatGPT’s evaluation**

Input: Letters 1 and 2.

Prompt: “Which of the two letters, Letter 1 or Letter 2, is more likely to be accepted for publication in a general medicine journal with an impact factor around 1-3?”

“Both letters present well-reasoned critiques. Letter 1, with its comprehensive approach and historical context, including the Kitasato anecdote, offers a compelling and thoughtful argument. Its personal and detailed nature may resonate with readers who value depth and cultural nuances. Letter 2 adopts a more structured and polished approach, focusing on clarity and flow. The inclusion of the proverb is a subtle way to connect with readers, adding cultural depth and enhancing its appeal. While Letter 1 provides a broader discussion with a personal touch, Letter 2 maintains a sharper focus on the core points, also with personal touch. Both are well-written, and their acceptance ultimately depends on the journal’s editorial preferences.”

## Discussion

The primary question was whether ChatGPT can “generate” appropriate human touch. The study also attempted to determine whether ChatGPT can “select” the most suitable human touch from multiple candidates, and whether such a ChatGPT-generated manuscript appealed to readers.

First, ChatGPT selected a suitable human touch from multiple candidates. Of the seven provided items, it chose items 2, 4, and 5 (Box 1). Interestingly, while I included two Nobel Prize-related anecdotes 6 and 7, ChatGPT “digested” them into a shorter phrase (see the footnote to Box 1 and the third paragraph of Letter 2, Box 2). It excluded items 1 and 3, which was appropriate, as they did not fully match the context. ChatGPT not only selected appropriately but also modified elements as needed.

Although reviewers were not explicitly asked to assess “anecdotes,” four spontaneously noted their appropriate use, saying they added relatability and appeal – three of the four preferred Letter 2. No other consistent or notable themes emerged from the reviewers’ comments. None specified which anecdotes they found most effective, as they were not explicitly asked to evaluate them. Thus, while it remains unclear whether ChatGPT used human touch better than a human, importantly, no reviewers reported that they felt that the anecdotes reduced the letter’s quality.

Second, this addresses the primary outcome of this study. ChatGPT generated a new human touch element without being provided with specific candidates. The proverb “Even a mountain crowded with trees gains nothing from lifeless trunks” roughly means “the more, the livelier the atmosphere.” Here, “lifeless trunks” implies something of no value, potentially suggesting that a coauthored clinic doctor holds no value, which might sound impolite. ChatGPT softened this with the phrase “an overstatement, of course.” Introducing a new proverb and then moderating its tone requires high-level writing skill. This interpretation is my own; reviewers did not comment on this specific point.

Setting aside the detailed algorithms of LLMs, selecting appropriate elements from candidates (“selection”) may be what such models are designed to do. Thus, selecting appropriate anecdotes may, in this sense, be a reasonable result. However, the generation of new anecdotes in a scientific writing context was not anticipated. Some may argue that ChatGPT did not generate the anecdotes “automatically on its own,” but instead likely depending on the context set by human input. Asking ChatGPT to “write a disagreement letter that incorporates human touch, freely selecting such elements on its own” might help to further examine this issue.

Third, the ChatGPT-generated letter was appealing. ChatGPT-generated manuscripts have been reported to be as compelling as those written by humans (4-6). This was also observed in the present study, even when ChatGPT was allowed to “select and create” human touch. As stated, this study did not attempt to test which letter was better. ChatGPT could have generated anecdotes, but the resulting letter might have been of low quality. In such a scenario, testing whether AI can generate anecdotes would have limited practical meaning. Thus, the purpose here was to establish a minimal benchmark – that the AI-generated letter, in the present setting, was not of low quality.

There are limitations that may affect the generalizability of these findings. First, this concerns the evaluation methods. The experiment was based on a single letter pair by a single author. I added another supplementary experiment, but it still involved letters by the same author. As this feasibility study suggests that the study design may be workable, future studies should expand the range of study materials. As to reviewers, they were asked to assess “appeal,” a broadly defined outcome. Given this and the limited sample size, these findings should be interpreted cautiously, and alternative interpretations remain possible. The reviewers were non-native English speakers. Native speakers might focus more on linguistic nuance (9). I intentionally chose non-native reviewers, as a considerable proportion of readers are also non-native speakers (10), although published texts are often edited by language experts.” Setting this aside, future studies should involve evaluators with varying levels of experience, including journal editors and language experts. More objective evaluation methods, including the use of standardized assessment scales, may further enhance evaluation quality. Regarding the ChatGPT-based evaluation, this approach is theoretically unsound, as LLMs may not be appropriate independent evaluators of text they generate. Therefore, this assessment should be regarded as informal information for readers. I also focused on the letter format rather than other manuscript types to test feasibility. The evaluation of human touch would likely not differ substantially between original articles and letters. Another limitation was that the impact of human touch on the manuscript’s overall value was not objectively measured. Once again, in this experiment, evaluation was required as a bottom line or prerequisite. However, no reviewers, although they were not specifically asked to assess this aspect, indicated that including human touch reduced the letter’s quality. Thus, this minimal requirement appears to have been met.

Some may consider “human touch” a peripheral issue in discussions of LLM use in medical writing. Indeed, to my knowledge, no journals or publisher guidelines currently address this topic (11). However, I believe that human touch provides a useful starting point for this discussion. It offers a useful lens through which we can examine more deeply the nature of human academic writing. I hope this pilot study will serve as a foundation for further discussion of LLMs’ place in academic writing.

Lastly, I tasked ChatGPT-5 with generating agreement and disagreement letters addressing this manuscript, involving newly generated human touch (Supplementary file(Supplementary Material)). As shown, ChatGPT again generated human touch without being provided with candidate elements. I leave it to the reader’s discretion whether these two letters are appealing.

I have deliberately refrained from including any anecdotes here. Perhaps ending the manuscript on this quiet note is itself a small gift to the reader – a broader, gently playful form of “human touch.”

Declaration of generative AI and AI-assisted technologies in the writing process During the preparation of this work the author used ChatGPT-5 in order to make the ChatGPT-generated manuscript (Letter 2), to generate agreement/disagreement letters addressing this manuscript (Supplementary), and to evaluate whether Letters 1 and 2 were likely to be accepted in a journal. I wrote this in the text and I take full responsibility for this.
