# Supplementary material for: Can artificial intelligence create human touch in medical writing? A pilot study
Source: Croat Med J. 2026 Apr;67(2):66–71. doi: 10.3325/cmj.2026.67.66 (PMC13176933; doi:10.3325/cmj.2026.67.66)
Supplement: Supplementary Material [file CroatMedJ_67_s004.pdf]

**Supplementary file: ChatGPT-5-generated Letters (agreement and disagreement)**

**Prompt 1 (Agreement Letter)**

I give you the whole manuscript. Write an agreement letter of approximately 200 words. You may, at your discretion, include points such as:

"The last wall distinguishing human-written from Generative AI-generated manuscripts may be the presence of 'human touch.' Medical papers should not be a mechanical record but a record of 'human endeavour.' Many preceding studies focused on the ability of ChatGPT in writing, but here the author, utilizing a ChatGPT-generated manuscript, prompted us to contemplate the real nature of human writing. This is a highly thought-provoking study."

You may also add anything else you find suitable.

**Important:** Please include some *human touch* (such as proverbs, old sayings, or anecdotes) that fit the context.

**Prompt 2 (Disagreement Letter)**

I give you the whole manuscript. Write a disagreement letter of approximately 200 words. You may, at your discretion, include points such as:

"The human touch may enrich a medical paper; however, a medical paper is fundamentally for transferring scientific observations. A study on this touch may illustrate some features of both human- and ChatGPT-writing, but this discussion may be more suitable for novels, poems, or essays. In scientific writing, what is written comes first, and how it is written comes second."

You may also add anything else you find suitable.

**Important:** Please include some *human touch* (such as proverbs, old sayings, or anecdotes) that fit the context.

**Agreement Letter**

The author’s study touches the very nerve of contemporary medical writing. The last wall distinguishing human-written from Generative AI-generated manuscripts may indeed be the presence of “human touch.” A medical paper should not be a mere mechanical record but a record of “human endeavor.” By asking ChatGPT to “select and create” anecdotes, the author highlights the subtle qualities that make writing resonate with readers. This is not about data alone, but about meaning, nuance, and engagement.

An old saying reminds us, “A story without a soul is like a body without breath.” The author’s experiment illustrates that ChatGPT, when prompted, can provide both body and breath, though the soul remains a matter for reflection. Many preceding studies examined ChatGPT’s technical writing ability, but this paper urges us to reconsider the essence of what separates a text that informs from one that also moves.

This is a highly thought-provoking study. It is a gentle but firm reminder that medical writing, though grounded in science, is still a human art.

**Disagreement Letter**

The author’s study is engaging, but I respectfully disagree with its central emphasis. The human touch may enrich a medical paper, yet science is judged primarily by its observations, not its embellishments. A study on anecdotes and proverbs may illustrate differences between human and ChatGPT writing, but such discussion may be better placed in essays or literary critique rather than medical journals. In scientific writing, what is written must come first, and how it is written second.

There is an old proverb: “Fine words butter no parsnips.” A paper rich in sayings may charm the reader, but it cannot replace sound evidence. Anecdotes may help in teaching or in bedside conversation, but when transferred into medical writing, they risk blurring the boundary between narrative and data.

Thus, while the author’s experiment is creative, I believe the true measure of scientific writing lies not in touches of style but in clarity, accuracy, and reproducibility. These must remain the pillars of our literature.

**With shorter prompts:**

In further experiments, one sentence prompt has been inputted, and ChatGPT-5 generated Letters (Agreement and Disagreement) with human touch, similarly. I omitted describing these Letters, which may simply complicate the issue.
